# Simultaneous reduction of flow and fraction of inspired oxygen (FiO_2_) versus reduction of flow first or FiO_2_ first in patients ready to be weaned from high-flow nasal cannula oxygen therapy: study protocol for a randomized controlled trial (SLOWH trial)

**DOI:** 10.1186/s13063-019-4019-7

**Published:** 2020-01-14

**Authors:** Min Chul Kim, Yeon Joo Lee, Jong Sun Park, Young-Jae Cho, Ho Il Yoon, Choon-Taek Lee, Jae Ho Lee, Eun Sun Kim

**Affiliations:** 1 Department of Internal Medicine, Seoul National University College of Medicine, Seoul, Republic of Korea; 2 Division of Pulmonary and Critical Care Medicine, Department of Internal Medicine, Seoul National University Bundang Hospital, Seongnam-si, Gyeonggi-do Republic of Korea; 3 Department of Hospital Medicine, Seoul National University Bundang Hospital, 82, Gumi-ro 173 Beon-gil, Bundang-gu, Seongnam-si, Gyeonggi-do 13620 Republic of Korea

**Keywords:** Adult, High-flow nasal cannula, Protocol, Weaning

## Abstract

**Background:**

High-flow nasal cannula (HFNC) oxygen therapy has been widely used in critically ill patients. Despite the effectiveness of HFNC as a treatment, optimal methods to withdraw HFNC after recovery from preexisting conditions have not been investigated to date. In this study, we will evaluate the safety and efficacy of simultaneous reduction of flow and fraction of inspired oxygen (FiO_2_) compared with sequential reduction of either flow first or FiO_2_ reduction first in patients with HFNC.

**Methods/design:**

This is a prospective, investigator-initiated, randomized controlled trial with three experimental intervention groups. A total of 100 adult patients receiving HFNC and satisfying weaning criteria will be enrolled and randomly assigned to one of the following groups: flow reduction (FR) first, FiO_2_ reduction (OR) first, or simultaneous reduction (SR). In the FR group, flow will be reduced first by 10 L/min/h. When it reaches 20 L/min, FiO_2_ will then be reduced by 0.1 /h until it reaches 0.3. In the OR group, the FiO_2_ will be gradually reduced first by 0.1 /h until it reaches 0.3, then flow will be reduced by 10 L/min until it reaches 20 L/min. Finally, in the SR group, both the flow and FiO_2_ will be gradually reduced simultaneously by 10 L/min and 0.1/h, respectively. Weaning will proceed only when patients satisfy the weaning criteria at every weaning point. When the HFNC weaning-off targets are reached (20 L/min and 0.3 for flow and FiO_2,_ respectively), the patient will be transferred to conventional oxygen therapy (mainly low-flow nasal prongs). The primary outcome is the time to successful weaning from HFNC for 24 h. Secondary outcomes will include the success or failure rate in weaning off HFNC and changes in arterial blood gas analyses, intolerance rate, length of hospital stay, and in-hospital mortality.

**Discussion:**

This study will be the first clinical trial to investigate the safety and efficacy of three different methods of weaning in adult patients receiving HFNC. Once this study is completed, we expect to be able to suggest the better strategy for withdrawal of HFNC based on the results.

**Trial registration:**

ClinicalTrials.gov, NCT03845244. Registered on 19 February 2019.

## Background

The heated and humidified high-flow nasal cannula (HFNC) has become an increasingly popular choice of therapy due to the potential complications of invasive ventilation [1, 2] and the frequent uncomfortable or life-threatening adverse effects that are produced in non-invasive ventilation (NIV) [3]. The HFNC allows modification of only two variables: the percentage of oxygen being delivered and the rate of gas flow [4]. Numerous well-designed studies have been conducted into the use of the HFNC in the treatment of critically ill patients, and HFNC is now widely used in the treatment of patients with various diagnoses including acute hypoxemic respiratory failure or acute respiratory distress syndrome (ARDS) [5, 6], and is used in post-extubation treatment [7–9], post-cardiothoracic surgery respiratory distress [10, 11], and respiratory compromise induced by heart failure [12, 13]. While studies are being conducted to determine both the optimal technique for HFNC delivery and the clinical setting in which it is most useful, the best strategy for weaning from HFNC remains unknown. It has also not been established at what point a patient should be considered stable enough to attempt to start withdrawing the HFNC.

Similarly, several limited studies have been conducted on strategies for weaning from NIV [14–16]. Lun et al. compared stepwise withdrawal to immediate withdrawal of NIV [16] and included 35 and 25 patients in the stepwise and immediate withdrawal groups, respectively. The rates of successful weaning were 74% for stepwise and 56% for immediate withdrawal, though the difference was not statistically significant (*p* = 0.139) [16]. Based on this result, the British Thoracic Society and Royal College of Physicians (BTS/RCP) recommend a protocol using stepwise reduction in NIV, which takes 4 days for weaning.

To the best of our knowledge, there is only one study into weaning protocols for the HFNC, which was conducted within a pediatric intensive care unit (ICU) [17]. The authors suggested a “holiday” weaning protocol. In the holiday protocol, patients scoring less than or equal to 6 qualified for a HFNC holiday trial on low-flow nasal cannula settings. Patients with a respirator assessment score (RAS) of 7 or 8 had HFNC flow decreased by half, and patients scoring more than 8 remained on current settings and were reassessed. If their RAS remained less than 6, a holiday patient stayed on a low-flow nasal cannula and continued traditional weaning based on oxygen saturations. If the RAS was 6–8, the patient was put on half the amount of flow and scoring was repeated. If the repeat RAS was greater than 8, holiday patients returned to initial settings. Out of 133 patients, 119 (89.5%) successfully weaned to low-flow nasal cannula within four holiday attempts, and 14 (10.5%) failed to be weaned. Though the holiday weaning method seems efficient, we have some concerns relating to its clinical application. First, this study was not designed for comparison of different methods of HFNC withdrawal. Second, there is a lack of detailed information about HFNC application and the risk of atelectotrauma. Last, this study included only children. This makes it hard to extrapolate to adults with HFNC. We are conducting this randomized controlled trial (RCT) to investigate the safety and efficacy of simultaneous reduction of the two variables compared to sequentially reducing either the flow first, or the fraction of inspired oxygen (FiO_2_) first for the withdrawal of the HFNC in adult patients who are stable and ready to be weaned.

## Methods/design

### Hypothesis

The time to weaning from the HFNC will be shorter in the simultaneous reduction group compared to the group having flow or FiO_2_ reduction first, without increasing the weaning failure rate.

### Study design and patients

This study is a prospective, investigator-initiated, randomized controlled study with three experimental groups. Patients aged over 18 years receiving respiratory support through a HFNC for any indication will be screened for study participation. Participants satisfying all weaning criteria, whether they are medical or surgical, will be prospectively recruited after providing written informed consent. The weaning criteria are as follows: patients who have recovered from their underlying condition, show no signs of respiratory distress (such as agitation, diaphoresis, or anxiety), with arterial pH ≥ 7.35, partial pressure of oxygen (SpO_2_) > 90% with FiO_2_ ≤ 0.5, respiratory rate ≤ 25 breaths/min, heart rate ≤ 120 beats/min, and systolic blood pressure (SBP) ≥ 90 mmHg. Patients will be excluded from the study if they have severe hypercapnia (pH < 7.25), respiratory arrest requiring tracheal intubation, cardiac arrest, acute coronary syndrome or life-threatening arrhythmias, or failure of more than two organs. Patients who have had recent trauma or burns to the neck and face, are pregnant, or who refuse to participate or cooperate in the study will be also excluded.

Informed consent will be obtained from the patients when they are ready to be weaned off the HFNC. However, if the patients cannot understand information about the study, remember the information, or communicate their decision by talking, using sign language, or any other means, they will be considered to be unable to make a decision. In that case, consent will be obtained from their families or the legal representatives.

### Randomization

A research coordinator will randomize the participants. An independent statistician will generate a list of random numbers. Eligible participants will be randomly assigned at a ratio of 1:1:1 to the flow reduction first (FR) group, the FiO_2_ reduction first (OR) group, or the simultaneous reduction (SR) group, in accordance with the predefined randomization list with a block size of 3. Information about the randomization will not be provided to the research supervisor, co-researcher, or patients. In the case of an emergency, co-researchers who are aware of the random assignments should avoid participating in future results analysis.

### Types of interventions

After randomization, the participants will undergo FR, OR, or SR strategies for weaning off the HFNC. In the FR group, flow will gradually be reduced by 10 L/min/h. When it reaches 20 L/min, FiO_2_ reduction will then begin at 0.1 /h until it reaches 0.3. In the OR group, FiO_2_ will gradually be reduced by 0.1 /h until it reaches 0.3. At this point, flow will be reduced by 10 L/min/h until it reaches 20 L/min. Finally, in the SR group, both flow and FiO_2_ will be gradually reduced simultaneously at a rate of 10 L/min and 0.1 /h, respectively, until they reach the HFNC weaning-off targets (20 L/min for flow and 0.3 for FiO_2_). Weaning will proceed only when the patient satisfies all the weaning criteria at every weaning point. When flow and FiO_2_ reach weaning-off targets, the patient will be transferred to conventional oxygen therapy, such as low-flow nasal prongs.

**Fig. 1:**
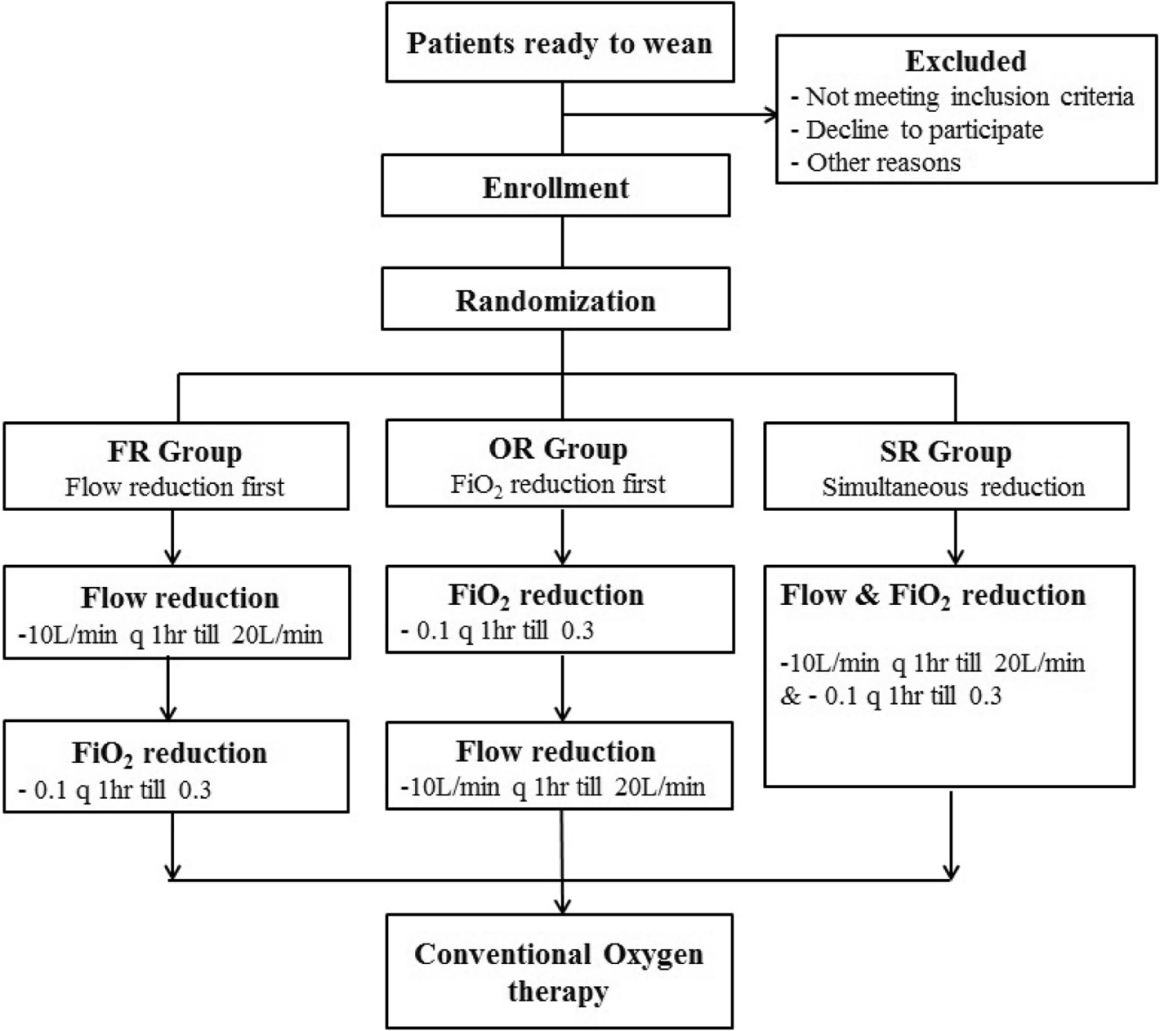
Study algorithm. FiO_2_, fraction of inspired oxygen; FR, flow reduction group; OR, FiO_2_ reduction group; SR, simultaneous reduction group

**Fig. 2:**
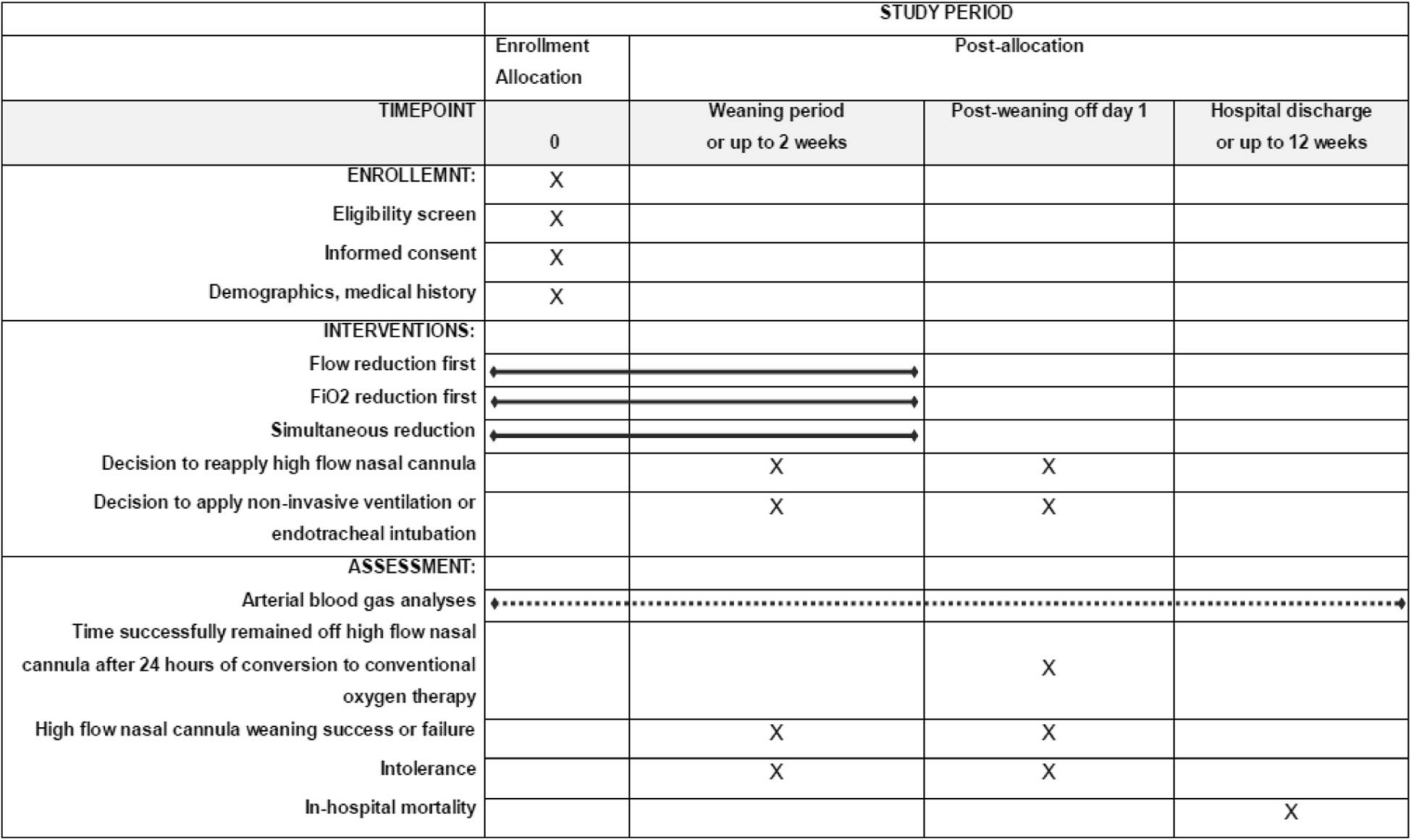
Schedule of enrollment, interventions, and assessments according to the Standard Protocol Items: Recommendations for Interventional Trials (SPIRIT) guideline

We have followed the Standard Protocol Items: Recommendations for Interventional Trials (SPIRIT) 2013 statement, which defines the standard protocol items for clinical trials [18] (see Additional file 1). The study algorithm is depicted in Fig. 1 and the SPIRIT schedule is shown in Fig. 2.

### Definitions

Weaning success is defined as a condition in which the patient meets all of the weaning criteria after 24 h of conversion to conventional oxygen therapy. A weaning failure is defined as the patients requiring re-application of the HFNC within 24 h of conversion to conventional oxygen therapy or needing NIV or endotracheal intubation during the HFNC weaning period or within 24 h of conversion to conventional oxygen therapy. If clinical improvement is not seen during follow up, NIV or intubation should be considered according to the clinician’s judgment. Immediate endotracheal intubation will be considered when there are two or more of the following conditions according to standard clinical practice: respiratory rate > 40 breaths/min, arterial pH < 7.2, PaO_2_ < 50 mmHg, signs of high work of breathing such as use of accessory muscle, abdominal paradox, need to secure the airway due to increased sputum, or altered mental status.

### Outcome measures

The primary outcome is time to successful weaning from HFNC after 24 h of conversion to conventional oxygen therapy. The secondary outcomes will include the success or failure to wean off HFNC (within and after 24 h of conventional oxygen therapy), changes of arterial blood gas analyses (PaO_2_, PaCO_2_, and pH), intolerance rate, length of hospital stay, and in-hospital mortality.

### Clinical and laboratory evaluations

A physical examination, laboratory evaluations, and medication reviews will be conducted before treatment. The laboratory evaluations including the arterial blood gas analysis (ABGA) will be performed based on the usual clinical practice, and additional examinations will be conducted if it is deemed necessary for treatment (irrespective of the study).

### Safety issues

The participants will be monitored for blood pressure, heart rate, respiratory rate, saturations, and electrocardiogram (ECG) during the study period. We will check for any clinical signs of deterioration and for patients’ discomfort at every time point. All adverse events related to the study will be recorded and followed up during the study period. Any serious adverse events will be reported to investigators and the ethics committee.

### Sample size calculation

It is difficult to calculate the target number of patients, because this is the first RCT evaluating the best strategy for weaning off the HFNC. Considering that 30 patients were compared in the NIV weaning protocol study, we estimate that a total of 90 participants, 30 in each group, would be needed. A total of 100 participants will be recruited in anticipation of a 10% dropout rate in each group based on the recent HFNC weaning study [17]. We have prepared for one interim analysis at the time when half of the subjects have completed the study.

### Statistical analyses

Statistical analyses will be performed on both a per-protocol (PP) and an intention-to treat (ITT) basis. For the PP analysis, all participants who complete the study will be included to evaluate the primary and secondary outcomes. For the ITT analysis, all participants enrolled and randomized to one of the three groups will be included. In the interim analysis, if the *P* value is < 0.003, we will stop the study early, accepting the significance of the outcome. If not, we will recruit the remaining half of the subjects. Sub-group analysis according to the respiratory failure type (hypoxemic or hypercapnic) is planned. Unless otherwise specified, continuous variables will be expressed as mean plus standard deviations or median and categorical variables as percentages. Student’s *t* test will be used to compare continuous variables; the chi-square or Fisher’s exact test will be used to compare categorical variables. Unless otherwise stated, all tests are two-sided and performed at the 0.05 significance level. Analyses will be performed using SPSS 20.0 (IBM Corp., Armonk, NY, USA).

### Data and safety monitoring

The paper data collection sheets and signed informed consents will be stored in a locked cabinet, and the electronic database will be stored on password-protected secure severs. Any unanticipated adverse events that occur during the study will be reported to the Institutional.

Review Board in accordance with the procedures of Seoul National University Bundang Hospital, Korea. Any proposed revised study procedure will be submitted to the Seoul National University Bundang Hospital Institutional Review Board for approval and to

ClinicalTrials.gov. The data will be kept confidential with only limited access to research investigators.

## Discussion

The HFNC is usually applied in critically ill patients. We therefore considered a study design that would minimize the risk to the patients. First, we adopted strict criteria for both patient inclusion and the weaning process to ensure patient safety. Second, we decided to compare three stepwise weaning protocols for HFNC without including immediate withdrawal from HFNC. The potential advantage of immediate withdrawal is a considerable shortening of the weaning process. However, risk of failure and the need for reinstitution of the HFNC or intubation is a major concern. In contrast to immediate withdrawal, stepwise weaning may minimize atelectotrauma and gradually increase respiratory muscle strength without the associated risk of atelectasis. Finally, the attending physician and nurse are requested to assess the patient’s condition within 30 min of changing the HFNC setting. In this study, several clinical outcomes will be compared between these three groups. After completion of the study, we hope to be able to suggest the best strategy for the withdrawal of the HFNC in adult patients.

There are several strengths of this study design. This will be the first study to evaluate HFNC weaning strategies in an adult population. It has been designed as a prospective RCT, which is one of the most powerful study designs. Furthermore, we will not conduct any medical tests or interventions other than HFNC weaning in this study in order to reflect the actual clinical situation. There are also some limitations of this study, namely, the small sample size and the fact that the study will be conducted at a tertiary referral hospital. In addition, we will adopt 1-h interval weaning, which is slower than the HFNC weaning process used in clinical practice. However, it was a necessary choice to ensure patient safety. Given the shortcomings of this first study, further additional studies will eventually be required on larger scale.

## Trial status

Protocol version 1.0 (approval date 12 November 2018). This trial started recruiting in January 2019. Recruitment is expected to conclude by December 2020.

## Supplementary information

**Additional file 1.** SPIRIT 2013 Checklist: Recommended items to address in a clinical trial protocol and related documents.

## Data Availability

Not applicable.

## Abbreviations

ABGA: Arterial blood gas analysis
ARDS: Acute respiratory distress syndrome
ECG: Electrocardiogram
FiO_2_: Fraction of inspired oxygen
FR: Flow reduction
HFNC: High-flow nasal cannula
ICU: Intensive care unit
ITT: Intention to treat
NIV: Non-invasive ventilation
OR: Reduction in fraction of inspired oxygen
PaCO_2_: Partial pressure of carbon dioxide
PaO_2_: Partial pressure of oxygen
PP: Per protocol
RCT: Randomized controlled trial
SBP: Systolic blood pressure
SD: Standard deviation
SNUBH: Seoul National University Bundang Hospital
SR: Simultaneous reduction

## References

[1] Antonelli M, Conti G, Rocco M, Bufi M, De Blasi RA, Vivino G (1998). A comparison of noninvasive positive-pressure ventilation and conventional mechanical ventilation in patients with acute respiratory failure. N Engl J Med.

[2] Ferrer M, Esquinas A, Leon M, Gonzalez G, Alarcon A, Torres A (2003). Noninvasive ventilation in severe hypoxemic respiratory failure: a randomized clinical trial. Am J Respir Crit Care Med.

[3] Carron M, Freo U, BaHammam AS, Dellweg D, Guarracino F, Cosentini R (2013). Complications of non-invasive ventilation techniques: a comprehensive qualitative review of randomized trials. Br J Anaesth.

[4] Helviz Y, Einav S (2018). A systematic review of the high-flow nasal cannula for adult patients. Crit Care.

[5] Rello J, Perez M, Roca O, Poulakou G, Souto J, Laborda C (2012). High-flow nasal therapy in adults with severe acute respiratory infection: a cohort study in patients with 2009 influenza A/H1N1v. J Crit Care.

[6] Frat JP, Thille AW, Mercat A, Girault C, Ragot S, Perbet S (2015). High-flow oxygen through nasal cannula in acute hypoxemic respiratory failure. N Engl J Med.

[7] Futier E, Paugam-Burtz C, Godet T, Khoy-Ear L, Rozencwajg S, Delay JM (2016). Effect of early postextubation high-flow nasal cannula vs conventional oxygen therapy on hypoxaemia in patients after major abdominal surgery: a French multicentre randomised controlled trial (OPERA). Intensive Care Med.

[8] Maggiore SM, Idone FA, Vaschetto R, Festa R, Cataldo A, Antonicelli F (2014). Nasal high-flow versus Venturi mask oxygen therapy after extubation. Effects on oxygenation, comfort, and clinical outcome. Am J Respir Crit Care Med.

[9] Hernandez G, Vaquero C, Colinas L, Cuena R, Gonzalez P, Canabal A (2016). Effect of postextubation high-flow nasal cannula vs noninvasive ventilation on reintubation and postextubation respiratory failure in high-risk patients: a randomized clinical trial. JAMA.

[10] Stephan F, Barrucand B, Petit P, Rezaiguia-Delclaux S, Medard A, Delannoy B (2015). High-flow nasal oxygen vs noninvasive positive airway pressure in hypoxemic patients after cardiothoracic surgery: a randomized clinical trial. JAMA.

[11] Parke R, McGuinness S, Dixon R, Jull A (2013). Open-label, phase II study of routine high-flow nasal oxygen therapy in cardiac surgical patients. Br J Anaesth.

[12] Roca O, Perez-Teran P, Masclans JR, Perez L, Galve E, Evangelista A (2013). Patients with New York Heart Association class III heart failure may benefit with high flow nasal cannula supportive therapy: high flow nasal cannula in heart failure. J Crit Care.

[13] Esquinas AM, Papadakos PJ (2014). High-flow nasal cannula supportive therapy in chronic heart failure: a partial or completed “CPAP-like effect”?. J Crit Care.

[14] Plant PK, Owen JL, Elliott MW (2000). Early use of non-invasive ventilation for acute exacerbations of chronic obstructive pulmonary disease on general respiratory wards: a multicentre randomised controlled trial. Lancet.

[15] Damas C, Andrade C, Araujo JP, Almeida J, Bettencourt P (2008). Weaning from non-invasive positive pressure ventilation: experience with progressive periods of withdraw. Rev Port Pneumol.

[16] Lun CT, Chan VL, Leung WS, Cheung AP, Cheng SL, Tsui MS (2013). A pilot randomized study comparing two methods of non-invasive ventilation withdrawal after acute respiratory failure in chronic obstructive pulmonary disease. Respirology.

[17] Betters KA, Hebbar KB, McCracken C, Heitz D, Sparacino S, Petrillo T (2017). A novel weaning protocol for high-flow nasal cannula in the PICU. Pediatr Crit Care Med.

[18] Chan AW, Tetzlaff JM, Altman DG, Laupacis A, Gotzsche PC, Krleza-Jeric K (2013). SPIRIT 2013 statement: defining standard protocol items for clinical trials. Ann Intern Med.

